# Editorial: The Microbiome in Hepatobiliary and Intestinal Disease

**DOI:** 10.3389/fphys.2022.893074

**Published:** 2022-04-13

**Authors:** Phillipp Hartmann

**Affiliations:** ^1^ Department of Pediatrics, University of California, San Diego, San Diego, CA, United States; ^2^ Division of Gastroenterology, Hepatology and Nutrition, Rady Children’s Hospital San Diego, San Diego, CA, United States

**Keywords:** Microbiome and dysbiosis, digestive diseases, Liver disease, Intestinal disease, microbiota

## Introduction

This Editorial provides a brief overview of the changes in the intestinal microbiome with focus on the bacterial microbiome in a wide range of diseases affecting the human digestive system and then highlights the specific articles of this Research Topic.

## Gut Microbiome Changes in Diseases Affecting the Human Digestive System

**FIGURE 1:**
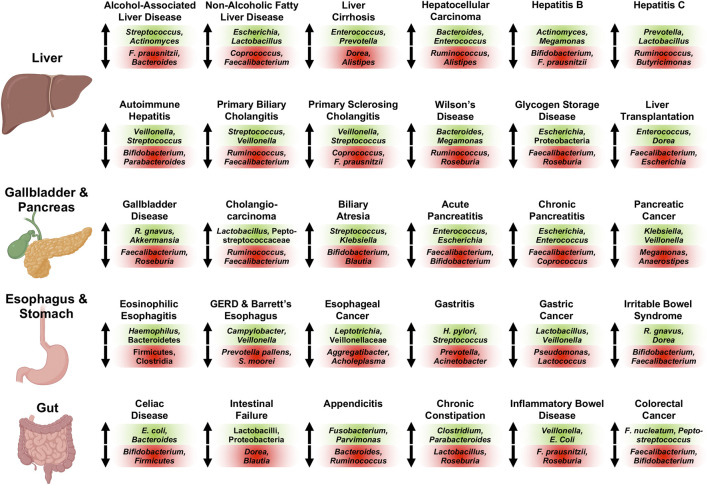
Representative associated intestinal bacterial microbiome changes across diseases of the human digestive system with focus on genera. Changes described here were observed in various publications for the respective conditions as detailed in the manuscript. Bacterial populations on green background are increased in abundance and populations on red background are decreased in abundance for the respective disease. Of note, *Veillonella* and Veillonellaceae, *Streptococcus*, *Lactobacillus* and Lactobacilli, *Escherichia (coli)*, *Enterococcus* (*faecalis* and *faecium*), and *Klebsiella* are oftentimes found at increased concentrations, whereas *Faecalibacterium (prausnitzii)*, *Ruminococcus* and Ruminococcaceae (except *Ruminococcus gnavus*), *Bifidobacterium*, *Roseburia*, and *Coprococcus* are frequently decreased in abundance in various digestive diseases. Created with a license from Biorender.com. *E. coli, Escherichia coli*; *F. nucleatum, Fusobacterium nucleatum;* *F. prausnitzii, Faecalibacterium prausnitzii*; GERD, gastroesophageal reflux disease; *R. gnavus, Ruminococcus gnavus; S. moorei, Solobacterium moorei.*

Essentially all diseases affecting the human digestive system are associated with significant increases and decreases of sub-populations of the gut microbiome compared with controls subjects; recurrent changes of the intestinal bacterial microbiome observed across 30 different conditions of the human digestive system are summarized in Figure 1. Briefly, *Streptococcus* (Llopis et al., 2016; Maccioni et al., 2020; Gao et al., 2021), *Actinomyces* (Ciocan et al., 2018; Maccioni et al., 2020; Gao et al., 2021), and *Rothia* (Ciocan et al., 2018; Maccioni et al., 2020) are increased, whereas *Faecalibacterium* (*prausnitzii*) (Gao et al., 2020a; Gao et al., 2020b; Maccioni et al., 2020) and *Bacteroides* (Puri et al., 2018; Gao et al., 2020a; Maccioni et al., 2020; Gao et al., 2021) are decreased in abundance in alcohol-associated liver disease (ALD). *Faecalibacterium* (*prausnitzii*) (Wong et al., 2013; Da Silva et al., 2018; Oh et al., 2020a) is also detected at diminished concentrations in non-alcoholic fatty liver disease (NAFLD) similar to *Coprococcus* (Zhu et al., 2013; Wang et al., 2016; Da Silva et al., 2018), whereas *Escherichia (coli)* (Zhu et al., 2013; Jiang et al., 2015; Oh et al., 2020a) and *Lactobacillus* (Raman et al., 2013; Jiang et al., 2015; Da Silva et al., 2018) are increased in NAFLD. Liver cirrhosis is associated with elevated intestinal levels of *Enterococcus (faecalis)* (Zhao et al., 2004; Chen et al., 2011; Bajaj et al., 2012), *Prevotella* (Qin et al., 2014; Chen et al., 2016; Shao et al., 2018; Ponziani et al., 2019; Zeng et al., 2020), *Clostridium* (Zhao et al., 2004; Chen et al., 2011; Bajaj et al., 2012; Qin et al., 2014; Heidrich et al., 2018; Shao et al., 2018), *Veillonella* (Qin et al., 2014; Chen et al., 2016; Shao et al., 2018; Oh et al., 2020a; Zeng et al., 2020), *Lactobacillus* (Qin et al., 2014; Heidrich et al., 2018; Shao et al., 2018; Ponziani et al., 2019; Zeng et al., 2020), *Atopobium* (Chen et al., 2016; Ponziani et al., 2019; Zeng et al., 2020), and *Streptococcus* (Qin et al., 2014; Shao et al., 2018; Ponziani et al., 2019; Oh et al., 2020a), and with reduced levels of *Dorea* (Bajaj et al., 2012; Oh et al., 2020a), *Alistipes* (Qin et al., 2014; Shao et al., 2018; Oh et al., 2020a), and *Subdoligranulum* (Bajaj et al., 2012; Qin et al., 2014; Shao et al., 2018). Gut microbiome changes in hepatocellular carcinoma (HCC) are similar to the ones identified in liver cirrhosis, indicating their relationship also on a microbial level. HCC is frequently linked to high intestinal amounts of *Bacteroides* (Ponziani et al., 2019; Huang et al., 2020; Zeng et al., 2020), *Enterococcus* (Ni et al., 2019; Ponziani et al., 2019; Xin et al., 2019), *Veillonella* (Ni et al., 2019; Zeng et al., 2020), and *Atopobium* (Ni et al., 2019; Zeng et al., 2020), and low amounts of *Ruminococcus* (Ren et al., 2019; Zeng et al., 2020), *Alistipes* (Ren et al., 2019; Huang et al., 2020), and *Bifidobacterium* (Ponziani et al., 2019; Xin et al., 2019). Hepatitis B is associated with increased *Actinomyces* (Wang et al., 2017; Yao et al., 2021), *Megamonas* (Wang et al., 2017; Joo et al., 2021), *Enterococcus* (*faecalis*) (Lu et al., 2011; Yao et al., 2021), *Veillonella* (Yang et al., 2020; Zeng et al., 2020; Yao et al., 2021), *Streptococcus* (Yang et al., 2020; Yao et al., 2021), and *Atopobium* (Zeng et al., 2020; Yao et al., 2021), and decreased *Bifidobacterium* spp. (Lu et al., 2011; Xu et al., 2012), *Faecalibacterium* (*prausnitzii*) (Lu et al., 2011; Yang et al., 2020), *Parabacteroides* (Wang et al., 2017; Yao et al., 2021), and *Ruminococcus* (Wang et al., 2017; Yao et al., 2021) in abundance, whereas patients with hepatitis C have been found enriched in *Prevotella* (Aly et al., 2016; Sultan et al., 2021a), *Lactobacillus* (Heidrich et al., 2018; Inoue et al., 2018), *Streptococcus* (Heidrich et al., 2018; Inoue et al., 2018), and *Veillonella* (Aly et al., 2016; Heidrich et al., 2018), and deplete of *Ruminococcus* (Aly et al., 2016; Mohieldeen et al., 2021) and *Butyricimonas* (Aly et al., 2016; Heidrich et al., 2018) compared with control subjects.

The various autoimmune liver diseases exhibit similar gut microbiome alterations: Stool samples of patients with autoimmune hepatitis are characterized by large quantities of *Veillonella* (Elsherbiny et al., 2020; Liwinski et al., 2020; Lou et al., 2020; Wei et al., 2020), *Streptococcus* (Elsherbiny et al., 2020; Liwinski et al., 2020; Wei et al., 2020), *Haemophilus* (Elsherbiny et al., 2020; Lou et al., 2020), and *Klebsiella* (Lou et al., 2020; Wei et al., 2020), and small quantities of *Bifidobacterium* (Lin et al., 2015; Liwinski et al., 2020), *Parabacteroides* (Elsherbiny et al., 2020; Lou et al., 2020; Wei et al., 2020), and Ruminococcaceae (Lou et al., 2020; Wei et al., 2020). Primary biliary cholangitis is associated with an elevated abundance of *Streptococcus* (Lv et al., 2016; Tang et al., 2018; Furukawa et al., 2020), *Veillonella* (Lv et al., 2016; Abe et al., 2018; Tang et al., 2018), *Klebsiella* (Lv et al., 2016; Tang et al., 2018), *Haemophilus* (Lv et al., 2016; Tang et al., 2018), and *Lactobacillus* (Tang et al., 2018; Furukawa et al., 2020), and diminished *Ruminococcus*/Ruminococcaceae (Lv et al., 2016; Furukawa et al., 2020) and *Faecalibacterium* (Tang et al., 2018; Furukawa et al., 2020), while primary sclerosing cholangitis is linked to enlarged proportions of *Veillonella* (Bajer et al., 2017; Kummen et al., 2017; Rühlemann et al., 2019; Cortez et al., 2020; Lapidot et al., 2021), *Streptococcus* (Sabino et al., 2016; Bajer et al., 2017; Rühlemann et al., 2019; Lapidot et al., 2021), and *Enterococcus* (Sabino et al., 2016; Bajer et al., 2017), as well as depressed proportions of *Coprococcus* (Bajer et al., 2017; Kummen et al., 2017; Rühlemann et al., 2019; Kummen et al., 2021), *Faecalibacterium prausnitzii* (Bajer et al., 2017; Lapidot et al., 2021), and Lachnospiraceae (Bajer et al., 2017; Kummen et al., 2017; Kummen et al., 2021; Lapidot et al., 2021). Enriched gut microbiota in Wilson’s Disease are *Bacteroides* (Geng et al., 2018; Cai et al., 2020) and *Megamonas* (Geng et al., 2018; Cai et al., 2020), whereas *Ruminococcus* (Cai et al., 2020) and *Roseburia* (Geng et al., 2018) are reduced. Glycogen storage disease shows overrepresented intestinal *Escherichia* (Colonetti et al., 2019; Ceccarani et al., 2020) and Proteobacteria (Colonetti et al., 2019; Ceccarani et al., 2020), and underrepresented *Faecalibacterium* (Colonetti et al., 2019; Ceccarani et al., 2020) and *Roseburia* (Colonetti et al., 2019; Ceccarani et al., 2020). Liver transplantation results in expansion of *Enterococcus* spp. (Wu et al., 2012; Annavajhala et al., 2019; Song et al., 2021a), *Dorea* (Bajaj et al., 2018; Annavajhala et al., 2019), *Blautia* (Sun et al., 2017; Bajaj et al., 2018; Song et al., 2021a), and *Streptococcus* (Bajaj et al., 2018; Annavajhala et al., 2019), and reduction of *Faecalibacterium (prausnitzii)* (Wu et al., 2012; Annavajhala et al., 2019; Lu et al., 2019; Song et al., 2021a), *Escherichia* (Bajaj et al., 2017; Sun et al., 2017; Bajaj et al., 2018), *Shigella* (Bajaj et al., 2017; Sun et al., 2017; Bajaj et al., 2018), and *Bifidobacterium* (Wu et al., 2012; Bajaj et al., 2018).

Gallbladder disease is linked to enriched *Ruminococcus gnavus* (Wang et al., 2020a; Zhang et al., 2021a) and *Akkermansia* (Liu et al., 2015; Zhang et al., 2021a), and depleted *Faecalibacterium* (Wu et al., 2013; Wang et al., 2020a), *Roseburia* (Wu et al., 2013; Keren et al., 2015; Zhang et al., 2021a), and *Prevotella 9* (Wang et al., 2020a; Zhang et al., 2021a). Cholangiocarcinoma is associated with enlarged fecal proportions of *Lactobacillus* (Jia et al., 2020; Zhang et al., 2021a) and Peptostreptococcaceae (Jia et al., 2020; Zhang et al., 2021a), and smaller proportions of *Ruminococcus* (Jia et al., 2020; Zhang et al., 2021a) and *Faecalibacterium* (Zhang et al., 2021a). The intestinal contributions of *Streptococcus* (Wang et al., 2020b; Song et al., 2021b) and *Klebsiella* (Wang et al., 2020b; Song et al., 2021b) are increased in biliary atresia, and those of *Bifidobacterium* (Wang et al., 2020b; Song et al., 2021b), *Blautia* (Wang et al., 2020b; Song et al., 2021b), and *Faecalibacterium* (Wang et al., 2020b; Song et al., 2021b) are decreased. The microbial changes occurring in acute and chronic pancreatitis compared with controls are similar: Acute pancreatitis is characterized by overrepresentation of *Enterococcus* (*faecalis*) (Zhu et al., 2019; Yu et al., 2020), *Escherichia* (*coli*) (Zhu et al., 2019; Yu et al., 2020), and Enterobacteriaceae (Tan et al., 2015; Zhu et al., 2019), and underrepresentation of *Faecalibacterium* (Zhu et al., 2019; Yu et al., 2020), *Bifidobacterium* (Zhu et al., 2019; Yu et al., 2020), and *Blautia* (Zhu et al., 2019; Yu et al., 2020), whereas chronic pancreatitis exhibits elevated fecal amounts of *Escherichia* (*coli*) (Savitskaia et al., 2002; Zhou et al., 2020a; Frost et al., 2020) and *Enterococcus* (*faecalis* and *faecium*) (Savitskaia et al., 2002; Frost et al., 2020), and lower amounts of *Faecalibacterium (prausnitzii)* (Jandhyala et al., 2017; Zhou et al., 2020a; Wang et al., 2020c; Frost et al., 2020), *Coprococcus* (Zhou et al., 2020a; Frost et al., 2020), *Subdoligranulum* (Zhou et al., 2020a; Wang et al., 2020c), and *Collinsella* (Zhou et al., 2020a; Wang et al., 2020c). Pancreatic cancer is associated with an expansion of *Klebsiella* (Ren et al., 2017; Pushalkar et al., 2018; Matsukawa et al., 2021), *Veillonella*/Veillonellaceae (Ren et al., 2017; Pushalkar et al., 2018; Half et al., 2019), *Parabacteroides* (Pushalkar et al., 2018; Matsukawa et al., 2021), and *Lactobacillus* (Ren et al., 2017; Matsukawa et al., 2021), as well as reduced *Megamonas* (Ren et al., 2017; Pushalkar et al., 2018), *Anaerostipes* (Ren et al., 2017; Pushalkar et al., 2018; Half et al., 2019), *Dorea* (Ren et al., 2017; Pushalkar et al., 2018), and *Firmicutes* (Ren et al., 2017; Matsukawa et al., 2021).

Eosinophilic esophagitis is linked to high abundances of *Haemophilus* (Harris et al., 2015; Hiremath et al., 2019) and Bacteroidetes (Benitez et al., 2015; Kashyap et al., 2019; Laserna-Mendieta et al., 2021), and low abundances of Firmicutes (Benitez et al., 2015; Kashyap et al., 2019) and Clostridia (Kashyap et al., 2019). Elevated concentrations of *Campylobacter* (*concisus*) (Macfarlane et al., 2007; Blackett et al., 2013; Deshpande et al., 2018; Snider et al., 2019) and *Veillonella* (Liu et al., 2013; Deshpande et al., 2018; Snider et al., 2018), and small quantities of *Prevotella pallens* (Snider et al., 2019; Kawar et al., 2021) and *Solobacterium moorei* (Zhou et al., 2020b; Kawar et al., 2021) can be detected in gastroesophageal reflux disease (GERD) and Barrett’s Esophagus. Esophageal adeno- and squamous cell carcinoma are associated with increased amounts of *Leptotrichia* (Lopetuso et al., 2020; Zhao et al., 2020), Veillonellaceae (Li et al., 2020a; Lopetuso et al., 2020; Zhao et al., 2020), and *Bifidobacterium* (Zhou et al., 2020b; Lopetuso et al., 2020; Zhao et al., 2020), and decreased amounts of *Aggregatibacter* (Chen et al., 2015; Zhao et al., 2020) and *Acholeplasma* (Chen et al., 2015; Zhao et al., 2020). Gastritis is characterized by enriched *Helicobacter pylori* (Parsons et al., 2017; Yang et al., 2019; Ndegwa et al., 2020) and *Streptococcus* (Li et al., 2009; Gao et al., 2018; Cui et al., 2019), and depleted *Prevotella* (Parsons et al., 2017; Cui et al., 2019; Ndegwa et al., 2020) and *Acinetobacter* (Parsons et al., 2017; Cui et al., 2019; Ndegwa et al., 2020). In gastric cancer, *Lactobacillus* (Qi et al., 2019; Wang et al., 2020d; Gantuya et al., 2020) and *Veillonella* (Castaño-Rodríguez et al., 2017; Qi et al., 2019; Wang et al., 2020d) are increased, whereas *Pseudomonas* (Wang et al., 2020d; Gantuya et al., 2020) and *Lactococcus* (Chen et al., 2019; Gunathilake et al., 2019; Wang et al., 2020d) are decreased. Irritable bowel syndrome is linked to overrepresented *Ruminococcus gnavus* (Rajilić–Stojanović et al., 2011; Rangel et al., 2015) and *Dorea* (*formicigenerans*) (Rajilić–Stojanović et al., 2011; Rangel et al., 2015; Maharshak et al., 2018), and underrepresented *Bifidobacterium* (*catenulatum*) (Malinen et al., 2005; Kerckhoffs et al., 2009; Rajilić–Stojanović et al., 2011) and *Faecalibacterium* (*prausnitzii*) (Carroll et al., 2012; Rangel et al., 2015; Maharshak et al., 2018).

Celiac disease is associated with enlarged proportions of *Escherichia coli* (Nadal et al., 2007; Collado et al., 2009; Schippa et al., 2010), *Bacteroides* (*fragilis* and *vulgatus*) (Nadal et al., 2007; Collado et al., 2009; De Palma et al., 2010; Schippa et al., 2010; Sánchez et al., 2012), and *Staphylococcus* (Collado et al., 2009; Sánchez et al., 2013), and contracted contributions of *Bifidobacterium* (Sanz et al., 2007; Collado et al., 2009; De Palma et al., 2010) and Firmicutes (Sánchez et al., 2013; Iaffaldano et al., 2018). The gut microbiome of patients with intestinal failure is enriched in *Lactobacillus*/Lactobacilli (Joly et al., 2010; Korpela et al., 2017) and Proteobacteria (Davidovics et al., 2016; Korpela et al., 2017), and diminished in *Dorea* (Huang et al., 2017; Piper et al., 2017) and *Blautia* (Huang et al., 2017; Piper et al., 2017). Acute appendicitis is characterized by elevated levels of *Fusobacterium* (Swidsinski et al., 2011; Guinane et al., 2013; Jackson et al., 2014; Zhong et al., 2014; Rogers et al., 2016), *Parvimonas* (Guinane et al., 2013; Jackson et al., 2014; Zhong et al., 2014; Rogers et al., 2016), *Campylobacter jejuni* (Campbell et al., 2006; Oh et al., 2020b), and *Gemella* (Guinane et al., 2013; Zhong et al., 2014), and reduced levels of *Bacteroides* (Swidsinski et al., 2011; Samuelsson et al., 2013; Zhong et al., 2014; Rogers et al., 2016), *Ruminococcus* (Samuelsson et al., 2013; Munakata et al., 2021), and *Faecalibacterium* (*prausnitzii*) (Swidsinski et al., 2011; Samuelsson et al., 2013). The gut microbiome signature of chronic constipation consists of large proportions of *Clostridium* (Zoppi et al., 1998; Zhu et al., 2014) and *Parabacteroides* (de Meij et al., 2016; Li et al., 2020b), and depressed amounts of *Lactobacillus* (Khalif et al., 2005; Moraes et al., 2016; Jomehzadeh et al., 2020) and *Roseburia* (Mancabelli et al., 2017; Li et al., 2020b). Stool analysis of patients with inflammatory bowel disease (IBD) frequently demonstrates overrepresentation of *Veillonella* (Gevers et al., 2014; Mottawea et al., 2016; Santoru et al., 2017; Schirmer et al., 2018) and *Escherichia coli* (Schwiertz et al., 2010; Sha et al., 2013; Gevers et al., 2014; Santoru et al., 2017), and underrepresentation of *Faecalibacterium* (*prausnitzii*) (Schwiertz et al., 2010; Joossens et al., 2011; Morgan et al., 2012; Kumari et al., 2013; Gevers et al., 2014; Machiels et al., 2014; Schirmer et al., 2018), and *Roseburia* (Morgan et al., 2012; Kumari et al., 2013; Rajilić-Stojanović et al., 2013; Gevers et al., 2014; Machiels et al., 2014). *Fusobacterium* (*nucleatum*) (Kostic et al., 2012; Ahn et al., 2013; Warren et al., 2013; Zeller et al., 2014; Gao et al., 2015; Mira-Pascual et al., 2015; Yu et al., 2017; Dai et al., 2018; Guo et al., 2018) is commonly enriched in colorectal cancer along with *Peptostreptococcus* (Wang et al., 2012; Ahn et al., 2013; Zeller et al., 2014; Gao et al., 2015; Yu et al., 2017), whereas *Faecalibacterium* (*prausnitzii*) (Balamurugan et al., 2008; Kostic et al., 2012; Guo et al., 2018) and *Bifidobacterium* (Mira-Pascual et al., 2015; Dai et al., 2018; Guo et al., 2018) are depressed in number.

Fungi, viruses, and other non-bacterial populations are also detected at aberrant proportions in disorders of the digestive system, e.g., the fungus *Candida albicans* is increased in ALD (Lang et al., 2020a; Hartmann et al., 2021), NAFLD (Demir et al., 2021), gastric cancer (Zhong et al., 2021), IBD (Sokol et al., 2017), and colorectal cancer. (Starý et al., 2020) Viruses have also been correlated with disease activity in alcoholic hepatitis (Jiang et al., 2020) and NAFLD (Lang et al., 2020b) among others. Archaea have been investigated as well, *Methanosphaera stadtmaniae* (Blais Lecours et al., 2014) has been found to be more abundant and *Methanobrevibacter smithii* (Ghavami et al., 2018) has been found to be depleted in the gut microbiome of patients with IBD.

## Disease Association Index

When evaluating the microbiome findings of these 30 disorders of the digestive system above, striking observations can be made: *Faecalibacterium* (*prausnitzii*) is associated with 16 of these conditions, and this bacterium is decreased in all of these 16 conditions. In contrast, the fecal abundance of *Veillonella* and Veillonellaceae is increased in 13 out of 13 diseases and that of *Streptococcus* is increased in 10 out of 10 conditions in which a robust association has been demonstrated. To evaluate how likely a microbial population is increased or decreased across diseases that it is associated with, a *Disease Association Index* (*DAI*) can be calculated by dividing the increased-decreased net value (= the number of diseases in which the microbial population is increased minus the number of diseases in which the microbial population is decreased) by the total number of conditions that the population has been associated with. E.g. the DAI for *Faecalibacterium* (*prausnitzii*) among the digestive diseases discussed above is −1 (= (0–16)/16); the DAI for *Veillonella* and Veillonellaceae as well as for *Streptococcus* is +1 (= (13–0)/13) and +1 (= (10–0)/10), respectively. The DAI ranges from −1 to +1; the higher the value the more likely the microbial population to be increased in the evaluated diseases, and the lower the more likely that the population is decreased in the analyzed conditions. The closer the DAI is to 0, the more ambivalent is the microbial population. A DAI of +0.6 or higher, and a DAI of −0.6 or lower indicates that the abundance of a microbe can be considered highly positively or negatively correlated with disease, respectively. *Ruminococcus* has a DAI of −0.6 (2 increases/8 decreases), indicating that it is predominantly decreased in digestive diseases (of note, the species *Ruminococcus gnavus* is responsible for both increased abundances of *Ruminococcus* in these diseases, see above). Additional notable DAIs: *Bifidobacterium* −0.8 (1 increase/9 decreases), *Lactobacillus*/Lactobacilli +0.78 (8/1), *Escherichia* (*coli*) +0.71 (6/1), *Enterococcus* (*faecalis*/*faecium*) +1 (7/0), *Dorea* −0.2 (2/3), *Prevotella* −0.2 (2/3), *Bacteroides* (*fragilis*/*vulgatus*) +0.2 (3/2), *Roseburia* −1 (0/5), and *Klebsiella* +1 (4/0).

Targeted repletion trials for bacterial populations that are predominantly decreased (such as repletion of *Faecalibacterium* (*prausnitzii*) in a murine colitis model), (Martín et al., 2014) or targeted elimination trials for populations that are predominantly increased (such as elimination of cytolytic *Enterococcus faecalis* via targeted bacteriophages in a murine model of alcohol-induced liver disease) (Duan et al., 2019) could be attempted in the future.

## Articles in this Research Topic

The articles in this Research Topic are very diverse and wide-ranging. Zheng et al. describe microbiome and metabolite differences in various autoimmune liver diseases (Zheng et al.). Warner et al. characterize the role of human beta defensin-2 in alcohol-induced liver injury in mice (Warner et al.). Chen et al. discuss the role of the microbiota in the pathogenesis of chemical-induced acute liver injury models in rodents and the protective use of probiotics herein (Chen et al.). Zhang et al. demonstrate that hepatic branch vagotomy results in decreased dysbiosis but increased hepatic steatosis and continued neuro-inflammation in murine cirrhosis secondary to carbon tetrachloride injections (Zhang et al.). Song et al. analyzed changes of the gut microbiome in patients with biliary atresia after liver transplantation (Song et al.). Chen et al. report microbiome and metabolite shifts in a mouse model of gallstone disease (Chen et al.). Rao et al. discuss microbiome changes in cholangiocarcinoma and related precancerous conditions (Rao et al.). Jihan et al. identified specific microbiome signatures in cancers affecting the esophagus, stomach, colon, and rectum (Wang et al.). Busing et al. review various changes in the microbiota and metabolism in eosinophilic esophagitis (Busing et al.). Wang et al. associate intratumor microbiome signatures with subtype, tumor stage, and survival in patients with esophageal carcinoma (Wang et al.). Hu et al. discuss alterations seen in the gut microbiota in food allergies and other allergic conditions (Hu et al.). Chu et al. use a variety of mouse models to induce gastritis and analyze the associated modulations of the intestinal microflora (Chu et al.). Sultan et al. discuss metabolite alterations associated with intestinal dysbiosis in IBD (Sultan et al.). Houshyar et al. review what is known about the role of fungi and archaea in IBD (Houshyar et al.). Zhao et al. evaluate the role of gut bacteria in a rat model of intra-abdominal hypertension (Zhao et al.). Montanari et al. detail the relationship of an pro-inflammatory state and gut dysbiosis, and the effects of diet and medications on the gut microbiota observed in disorders of inborn errors of metabolism (Montanari et al.). Lastly, Li et al. introduce *Amadis*, a comprehensive, manually curated database that documents experimentally supported microbiota-disease associations (Li et al.).
